# Research progress and strategy of FGF21 for skin wound healing

**DOI:** 10.3389/fmed.2025.1510691

**Published:** 2025-03-31

**Authors:** Shisheng Lin, Lu Tang, Nuo Xu

**Affiliations:** ^1^College of Life and Environmental Sciences, Wenzhou University, Wenzhou, Zhejiang, China; ^2^School of Pharmaceutical Science, Wenzhou Medical University, Wenzhou, Zhejiang, China

**Keywords:** FGF21, skin wounds, trans-dermal drug delivery system, trans-dermal method, cell penetrating peptide

## Abstract

Fibroblast Growth Factor 21 (FGF21), a pivotal member of the fibroblast growth factor family, exhibits multifaceted biological functions, including the modulation of pro-inflammatory cytokines and metabolic regulation. Recent research has revealed that in impaired skin tissues, FGF21 and its receptors are upregulated and play a significant role in accelerating the wound healing process. However, the clinical application of FGF21 is severely limited by its short *in vivo* half-life: this factor is often degraded by enzymes before it can exert its therapeutic effects. To address this limitation, the transdermal drug delivery system (TDDS) has emerged as an innovative approach that enables sustained drug release, significantly prolonging the therapeutic duration. Leveraging genetic recombination technology, research teams have ingeniously fused FGF21 with cell-penetrating peptides (CPPs) to construct recombinant FGF21 complexes. These novel conjugates can efficiently penetrate the epidermal barrier and achieve sustained and stable pharmacological activity through TDDS. This review systematically analyzes the potential signaling pathways by which FGF21 accelerates skin wound repair, summarizes the latest advancements in TDDS technology, explores the therapeutic potential of FGF21, and evaluates the efficacy of CPP fusion tags. The manuscript not only proposes an innovative paradigm for the application of FGF21 in skin injury treatment but also provides new insights into its use in transdermal delivery, marking a significant step toward overcoming existing clinical therapeutic challenges. From a clinical medical perspective, this innovative delivery system holds promise for addressing the bioavailability issues of traditional FGF21 therapies, offering new strategies for the clinical treatment of metabolism-related diseases and wound healing. With further research, this technology holds vast potential for clinical applications in hard-to-heal wounds such as diabetic foot ulcers and burns.

## 1 Progress of FGF21 in the field of skin wounds

### 1.1 Overview of skin wound healing

The skin serves as a crucial barrier between the internal body systems and the external environment, playing a vital role in maintaining homeostasis (1). Skin damage compromises this protective barrier, potentially exposing the body to pathogen invasion risks and substantial fluid loss. Therefore, prompt restoration of skin integrity following injury represents the most effective approach to reestablish internal equilibrium, combat infections, and prevent fluid loss and electrolyte imbalance (2). Skin wound healing constitutes a complex and precisely regulated biological process that occurs through four overlapping phases: hemostasis/inflammation, proliferation, and remodeling (3). Disruption in any of these processes may result in impaired wound healing and the development of chronic wounds, including diabetic foot ulcers and pressure injuries, which present significant clinical challenges and affect approximately 1–2% of the global population (4).

Following skin injury, necrotic cells and disrupted tissues release damage-associated molecular patterns (DAMPs) and pathogen-associated molecular patterns (PAMPs), which trigger the activation of innate immune responses. These molecular patterns stimulate resident immune cells, including mast cells, T cells, and macrophages. Specifically, activated macrophages engage in downstream inflammatory signaling pathways by binding to their cognate receptors (5), thereby initiating the release of pro-inflammatory mediators. These pro-inflammatory factors induce vasodilation and facilitate the adhesion and transmigration of neutrophils and monocytes (6). Neutrophil recruitment to the wound site is mediated through chemoattractants such as interleukin-1 (IL-1), tumor necrosis factor-*α* (TNF-α), and bacterial endotoxins like lipopolysaccharide (LPS). Upon activation, neutrophils respond to inflammatory signals and pathways, including NF-κB, by secreting pro-inflammatory cytokines such as TNF-*α*, IL-1β, and IL-8 (7). Additionally, neutrophils clear necrotic tissue and pathogens via phagocytosis and the release of reactive oxygen species (ROS), antimicrobial peptides, and proteolytic enzymes (8). However, excessive neutrophil infiltration may have detrimental effects on tissue repair and delay wound healing (9). During the intermediate healing phase, neutrophils undergo reverse transmigration back into the bloodstream or undergo programmed cell death and subsequent clearance by macrophages (10).

Monocytes migrate to the inflammatory site during the later stages of the inflammatory response, where they either directly participate in phagocytic immune responses or differentiate into inflammatory macrophages (11). It is important to distinguish monocyte-derived macrophages from the conventional dendritic cells residing in the skin, including Langerhans cells (LCs) in the epidermis and dermal macrophages in the dermis (12). Emerging evidence indicates that the population of LCs in the epidermal layer significantly increases during the initial phases of wound healing and in healing diabetic foot ulcers (DFUs) compared to non-healing DFUs, suggesting a potential therapeutic role of LCs in diabetic wound management (13). Due to their remarkable plasticity, macrophages exhibit more diverse functions than monocytes and assume the phagocytic roles previously performed by polymorphonuclear neutrophils (PMNs) and monocytes. This plasticity enables their functional transition from pro-inflammatory phenotypes in the early healing stages to anti-inflammatory/reparative phenotypes during the proliferative phase (14). Wound macrophages originate from two primary sources: tissue-resident macrophages, which constitute a minor proportion, and monocyte-derived macrophages, which predominate and can rapidly expand in response to inflammatory stimuli (15).

Conventional wound management approaches encompass a range of surgical interventions, non-surgical therapies, and pharmacological treatment regimens for skin wound repair (16). While surgical debridement remains the gold standard in wound management due to its clinical efficacy (17), it can induce significant patient discomfort. Pharmacological interventions have emerged as promising alternatives to promote wound healing while minimizing patient distress. Among these, growth factor-based therapies (e.g., FGF21, EGF, and PDGF) represent a promising therapeutic approach. However, several limitations, including their short biological half-life, receptor desensitization issues, and reduced efficacy in metabolic-impaired microenvironments, necessitate the development of novel therapeutic strategies (18, 19).

### 1.2 Overview of FGF21

Fibroblast growth factor 21 (FGF21), fibroblast growth factor 19 (FGF19), and fibroblast growth factor 23 (FGF23) constitute the FGF19 subfamily, exhibiting significant structural homology (20). The human FGF21 gene, located on chromosome 19, encodes a 209-amino acid precursor protein. Following synthesis, the precursor undergoes proteolytic cleavage of its signal peptide, yielding a mature protein comprising 181 amino acids. Notably, the human FGF21 mature protein shares 75% sequence homology with its murine counterpart (21). FGF21, a recently identified metabolic regulator, demonstrates predominant expression in adipocytes, hepatocytes, and pancreatic *β*-cells, where it plays a pivotal role in modulating lipid and carbohydrate metabolism (22).

The biological activities of FGFs are primarily mediated through fibroblast growth factor receptors (FGFRs). However, FGFs cannot directly interact with FGFRs, requiring co-activator binding for effective activation of FGFR signaling pathways (23). Specifically, FGF21 must first interact with *β*-klotho, a transmembrane protein, before forming a ternary complex with FGFRs to initiate downstream signaling cascades (24). This molecular interaction is particularly significant in adipose tissue, liver, and pancreatic cells, where both FGF21 and *β*-klotho are predominantly co-expressed (25). Although FGF21 is principally synthesized and secreted by hepatic and adipose tissues (26), however, emerging experimental evidence has revealed that FGF21 exhibits significant upregulation in injured skin tissues (27). Following skin injury, the expression levels of both FGF21 and its co-receptor *β*-klotho demonstrate continuous elevation, reaching up to 2.2-fold and 4-fold increases, respectively, compared to baseline levels (28). Despite the observed high expression of FGF21 during cutaneous wound healing, its precise mechanistic role in promoting tissue repair remains incompletely understood. In wound healing assays, Western blot analysis has demonstrated that FGF21 expression is modulated by Wnt pathway activators. These investigations have revealed that FGF21 facilitates fibroblast migration and enhances skin wound repair processes. Notably, these studies have identified a reciprocal regulatory loop between FGF21 and Wnt signaling pathways, mediated through *β*-catenin in dermal fibroblasts (29). The interplay between FGF21 and Wnt signaling during cutaneous wound healing has been further corroborated by subsequent research (30). Persistent inflammation is a critical impediment to cutaneous wound repair, significantly hindering the healing process. FGF21 plays a pivotal role in modulating inflammatory responses, as evidenced by the development of various inflammatory disorders in FGF21-deficient conditions, including non-alcoholic fatty liver disease (NAFLD) and pancreatitis (31, 32). FGF21 has demonstrated potent anti-inflammatory properties across multiple pathological models. Experimental studies have shown that FGF21 can mitigate free fatty acid-induced nephritis in mice, reduce monosodium glutamate (MSG)-induced hepatic steatosis in obese mice, alleviate high-fat diet (HFD)-induced NAFLD, and attenuate lipopolysaccharide (LPS)-induced sepsis-associated inflammation (33–36). In the context of corneal epithelial wound healing, FGF21 has been shown to enhance epithelial repair while significantly reducing the levels of pro-inflammatory cytokines, including TNF-*α*, IL-6, and IL-1β, in diabetic mouse epithelium (37). Beyond its cellular functions, FGF21 interacts with multiple signaling pathways, such as Nrf2 and SIRT1, which regulate metabolism and fibrosis. Investigating these pathways could provide deeper insights into FGF21’s tissue-specific effects and its potential role in skin wound healing, an area that remains poorly understood.

## 2 Signal pathways of FGF21 promoting skin wound healing

The wound-healing effects of FGF21 appear to be mediated through multiple signaling pathways, with emerging evidence implicating the Nrf2-NFκB axis, JNK signaling, and SIRT1 pathways in this process (27, 38–40). These pathways collectively influence various stages of wound repair, including the modulation of inflammatory responses, promotion of cellular migration and recruitment, and regulation of the wound microenvironment to facilitate tissue regeneration. Cutaneous wound healing is a complex biological process that necessitates the coordinated interplay of multiple cell types, particularly involving the proliferation, migration, and differentiation of epithelial keratinocytes (41), macrophages (42), and fibroblasts (43), among others. These cellular processes are tightly regulated through intricate interactions with various molecular mediators, including cytokines, antimicrobial peptides, and growth factors (44). However, the precise mechanistic interplay between these signaling pathways and their integration into the broader wound healing process remains an area of active investigation. Understanding these mechanisms could provide valuable insights into developing therapeutic strategies for impaired wound healing. In the following sections, we will elaborate on the key signaling pathways through which FGF21 facilitates skin wound repair.

Nuclear factor erythroid 2-related factor 2 (Nrf2), a crucial transcriptional regulator, plays a central role in orchestrating cellular defense mechanisms against oxidative stress and modulating inflammatory responses (45). The biological activity of Nrf2 is mediated through its binding to antioxidant response elements (ARE) in the promoter regions of target genes, thereby regulating their expression. A prominent example is the heme oxygenase-1 (HO-1) gene, whose activation by Nrf2 confers significant antioxidant, anti-inflammatory, and anti-apoptotic properties to the cell (46). Extensive research has elucidated the therapeutic potential of FGF21 in diabetic wound healing. Experimental findings demonstrate that FGF21 administration significantly accelerates wound closure and enhances the overall healing process. Mechanistic investigations through Western Blot analysis reveal that FGF21 activates the Nrf2 signaling pathway, subsequently upregulating key antioxidant enzymes including HO-1 and NQO1, thereby ameliorating oxidative stress at the wound site (47). Furthermore, FGF21 treatment markedly reduces the production of pro-inflammatory cytokines such as TNF-*α*, IL-1β, and IL-6, potentially through its inhibitory effect on the NF-κB signaling pathway (48).

In macrophage-specific studies, FGF21 administration significantly downregulates the expression of inflammatory cytokines in LPS-induced macrophages while simultaneously enhancing the expression of HO-1 and Nrf2. These findings collectively demonstrate that FGF21 exerts its antioxidant and anti-inflammatory effects primarily through modulation of the Nrf2-NF-κB signaling pathway in macrophages (49).

In conclusion, FGF21 emerges as a pivotal regulator in cutaneous wound repair, with its therapeutic efficacy predominantly mediated through the Nrf2-NF-κB signaling pathway.

The c-Jun N-terminal kinase (JNK) serves as a crucial stress sensor cascade that integrates cellular responses to both stress and inflammatory stimuli (50). Recent studies have demonstrated that FGF21 plays a significant role in tissue repair processes, including skin wound healing. In this study, mice treated with FGF21 exhibited increased activation of phosphorylated JNK (p-JNK) compared to untreated controls, suggesting that FGF21 may enhance fibroblast migration and proliferation through the JNK signaling pathway (51). This finding is supported by previous research showing that FGF21 promotes fibroblast motility and accelerates wound closure in animal models (28). Together, these results indicate that FGF21 represents a potential therapeutic mechanism for promoting skin wound healing by modulating fibroblast behavior and JNK signaling activity. Further investigations have been conducted on the therapeutic potential of a fusion protein composed of FGF21 and elastin-like peptide (ELP) in diabetic wound healing models. It was demonstrated that the ELP-FGF21 fusion protein markedly improved wound healing quality through multiple mechanisms. Specifically, the levels of inflammatory factors were reduced, collagen synthesis and deposition were enhanced, and vascular network formation was promoted, all of which are essential for effective skin wound healing. These beneficial effects were attributed to the activation of the JNK signaling pathway by ELP-FGF21 (52). Additional evidence has been provided that fibroblast migration is significantly enhanced by FGF21 treatment through the upregulation of JNK signaling pathway activity (27). The activation of the JNK signaling pathway has been identified as a crucial mechanism for facilitating cellular migration, which represents a vital step in the wound healing cascade.

Sirtuin 1 (SIRT1), a NAD + -dependent protein, is characterized by its dual enzymatic activities as both a deacetylase and an ADP-ribosyltransferase (53). This multifunctional protein has been implicated in various cellular processes, including oxidative stress response, cell growth regulation, differentiation modulation, and apoptosis mediation (54). Emerging evidence has identified SIRT1 as a critical regulator in keratinocyte differentiation during the re-epithelialization process, with additional implications in skin aging and barrier function regulation (55, 56). More importantly, FGF21 has been demonstrated to play a pivotal role in facilitating the migration and differentiation of both dermal and epidermal cells during physiological wound healing, primarily through mechanisms involving SIRT1-dependent autophagy pathways (39). The recruitment and activation of fibroblasts have been demonstrated to play a crucial role in wound healing, primarily through regulation of granulation tissue formation, angiogenesis, and epidermis regeneration (57). Epidermal SIRT1 deficiency has been shown to inhibit epidermal regeneration and dermal stroma recovery, while also reducing the production of cytokines (IL-1β) and the recruitment and activation of macrophages, neutrophils, mast cells, and fibroblasts. Both *in vivo* and *in vitro* experiments have demonstrated that SIRT1 enhances wound healing capacity and moderately promotes cellular proliferation (58). Specifically, corneal epithelial wound healing was found to be promoted by SIRT1 through mechanisms that enhance corneal epithelial cell migration (59). These research findings collectively support the hypothesis that SIRT1 may be targeted therapeutically to promote cutaneous wound healing.

The wound healing process is characterized by several overlapping phases that are meticulously coordinated through the concerted actions of multiple cell types, including macrophages, neutrophils, T regulatory (T-reg) cells, and fibroblasts (60). Among these cellular components, macrophages have been identified as central regulators that orchestrate critical aspects of wound repair, specifically through their involvement in inflammation modulation, angiogenesis regulation, and tissue remodeling processes (61). Macrophages are characterized by two distinct phenotypes: the M1 phenotype, which is predominantly associated with pro-inflammatory responses, and the M2 phenotype, which is primarily involved in anti-inflammatory processes (62). The M1 phenotype is typically identified by its pro-inflammatory activity, marked by the secretion of cytokines including TNF-*α*, IL-1*β*, and IL-6. In contrast, the M2 phenotype is distinguished by its secretion of anti-inflammatory mediators such as transforming growth factor-β (TGF-β), IL-10, and IL-4, which are crucial for inflammation resolution and tissue repair (63, 64). Numerous compounds, including phosphatidylserine liposomes (65), quercetin (66), insulin (67), and vascular endothelial growth factors (68), have been demonstrated to enhance M2 macrophage polarization, thereby accelerating wound healing. The transition from M1 to M2 phenotype macrophages is recognized as a critical process in wound healing (69). Accumulating evidence suggests that FGF21 plays a regulatory role in macrophage polarization, facilitating the conversion from M1 to M2 phenotype macrophages. Experimental studies have revealed that FGF21 treatment modulates macrophage polarization in THP-1-derived macrophages, protecting against ox-LDL-induced apoptosis (70). Additional research indicates that FGF21 supplementation reduces LPS-induced M1 macrophage marker expression and promotes M2 phenotype features through the AMPK signaling pathway, as evidenced by diminished effects following AMPK inhibitor administration (71).

Although direct evidence linking FGF21-mediated macrophage polarization to skin wound healing remains to be established, macrophages represent a potential therapeutic target for FGF21 in promoting cutaneous wound healing.

**Figure 1:**
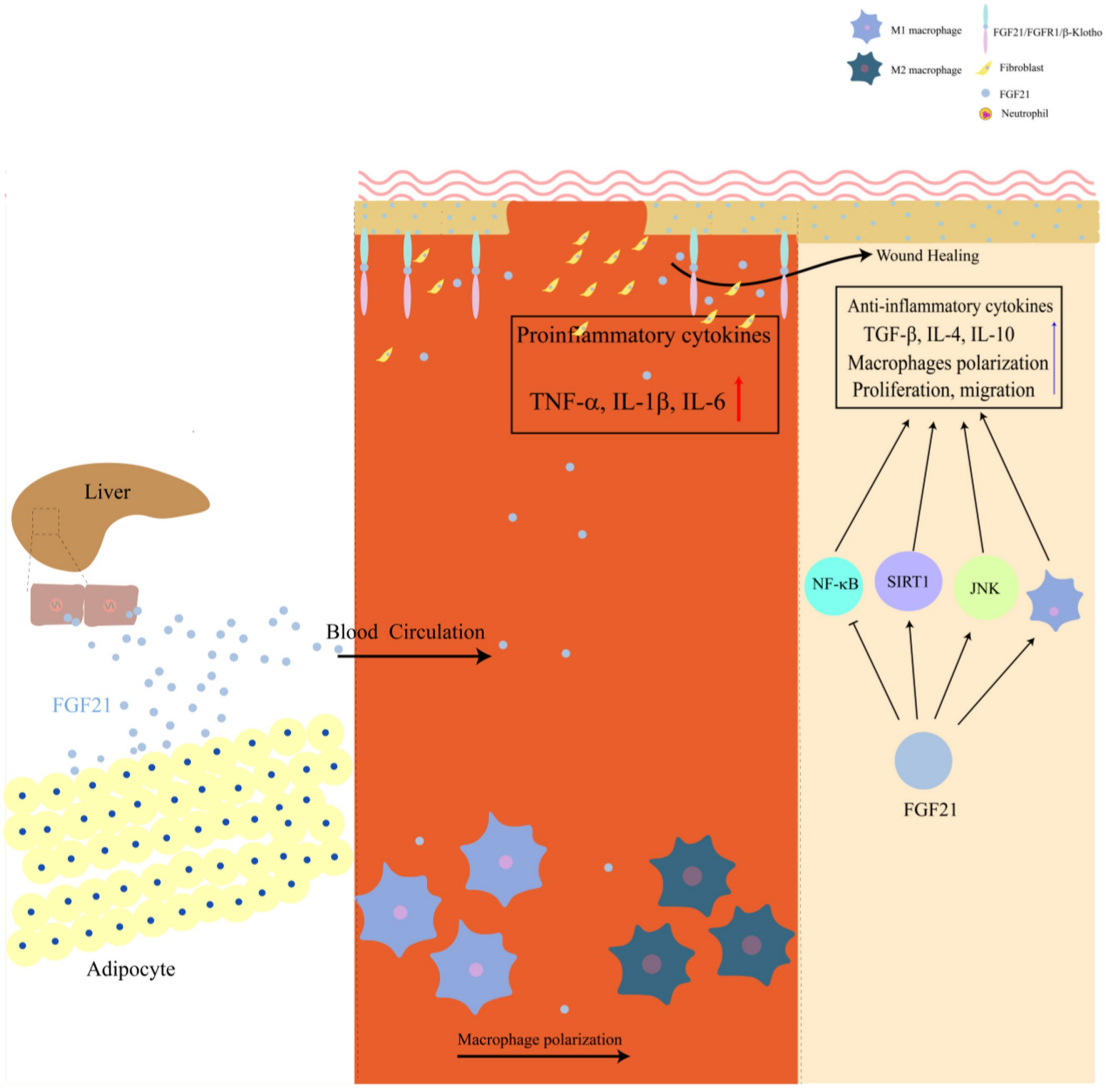
Mechanisms of FGF21 in promoting wound repair.

In summary, FGF21 exerts multifaceted therapeutic effects on wound healing through the modulation of multiple signaling pathways, including Nrf2-NF-κB, JNK, and SIRT1-mediated mechanisms. These pathways collectively contribute to enhanced cellular migration, reduced inflammation, and attenuated oxidative stress during the wound healing process. Evidence suggests that FGF21 may also regulate macrophage polarization by promoting the phenotypic transition from pro-inflammatory M1 to anti-inflammatory M2 macrophages, thereby potentially accelerating cutaneous wound healing. The pleiotropic functions of FGF21 establish a novel therapeutic paradigm for wound repair, offering promising clinical applications (Figure 1).

## 3 Current research status of trans-dermal drug delivery system for FGF21

### 3.1 Overview of trans-dermal delivery system

The skin, constituting approximately one-third of human tissue, encompasses a surface area of about two square meters (72). This complex organ is composed of three distinct layers: the epidermis, dermis, and subcutaneous tissue. As a primary delivery organ for dermatological therapeutics, the skin allows drug penetration primarily through passive diffusion across the stratum corneum into the inter-follicular region (73). Following penetration, drugs subsequently diffuse to target tissues to exert their pharmacological effects. However, this process is restricted by the skin’s epidermal barrier, which limits the passage of molecules exceeding 500 Da, thereby imposing significant constraints on the therapeutic application of high molecular weight compounds.

Since the landmark discovery of insulin in 1921, remarkable progress has been achieved in the development of therapeutic proteins and peptides, encompassing antibodies, hormones, cytokines, and vaccines (74). To date, over 240 protein and peptide-based therapeutics have received FDA approval for disease treatment (75). Unlike small-molecule drugs, protein therapeutics exhibit high specificity and complex biological functions due to their intricate structural configurations.

Despite the development of various non-injectable drug delivery strategies, including oral, transdermal, nasal, mucosal, pulmonary, and rectal routes, significant challenges persist. Oral delivery, the most commonly employed strategy, is particularly hindered by gastrointestinal barriers characterized by gastric pH variations, proteolytic enzyme activity, and inefficient intestinal absorption mechanisms (76). Consequently, intravenous administration remains the predominant clinical route for protein and peptide delivery (77), despite its associated limitations of patient discomfort, high costs, and logistical challenges.

The Transdermal Drug Delivery System (TDDS) has emerged as a prominent non-invasive method for the systemic administration of therapeutic agents through the skin. This innovative system has demonstrated significant clinical utility across diverse therapeutic areas, including pain management, hormone replacement therapy, and the treatment of cardiovascular and central nervous system disorders (78–80). One of the key advantages of TDDS lies in its ability to bypass the gastrointestinal tract, thereby eliminating first-pass metabolism and its associated limitations on drug bioavailability. This unique delivery mechanism ensures that therapeutic agents are absorbed systemically without interference from gastric pH variations, enzymatic degradation, or intestinal microbiota. Specifically, TDDS facilitates drug absorption through the skin’s epidermal and dermal layers, where agents subsequently enter the microcirculation and systemic circulation at controlled, sustained rates (81). Furthermore, TDDS has exhibited remarkable versatility in enabling the permeation of diverse therapeutic agents across the skin barrier, including lipid-based compounds, proteins, nucleic acids, and collagen-based formulations. This systemic delivery approach offers distinct advantages over traditional administration routes, particularly for compounds that are susceptible to gastrointestinal degradation or rapid hepatic metabolism.

In recent years, TDDS has emerged as a pivotal strategy in disease prevention and therapeutic intervention. Nevertheless, the intrinsic barrier properties of the skin continue to present significant challenges to TDDS development and optimization. Notably, substantial advancements have been achieved in recent decades across three principal domains of TDDS technology: physical, chemical, and biological enhancement methods.

### 3.2 Physical approach

Physical enhancement methods have demonstrated significant efficacy in improving skin permeability for drug and biomolecule delivery through the application of various external energy modalities, including electrical, mechanical, and physical stimuli (82). These techniques offer dual advantages: ensuring rapid and reliable drug penetration through the skin barrier while simultaneously enhancing the therapeutic efficacy of delivered agents (83, 84).

#### 3.2.1 Ultrasound-induced penetration

In ultrasound-mediated transdermal drug delivery, the penetration of therapeutic agents is typically facilitated through aqueous channels created by high-frequency sound waves (ranging from 20 kHz to 16 MHz). The effectiveness of drug delivery is known to be enhanced when ultrasound is applied within an optimal frequency range (83). Specifically, lower ultrasound frequencies have been shown to promote the formation of water pathways in the stratum corneum, thereby accelerating drug permeation (85). Additionally, localized heating of the skin area is induced by ultrasound, generating a thermal effect that further promotes drug penetration. It should be noted that the efficacy of ultrasound-mediated delivery is not influenced by drug properties such as solubility, dissociation constant, or hydrophilicity, as demonstrated with compounds including mannitol and insulin.

Innovative nanoparticle formulations, such as perfluoropropane (C3F8)-loaded and polyethylenimine (PEI)-doped poly(lactic-co-glycolic acid) nanoparticles (CPPPNBs), have been developed to enhance drug delivery. Specifically, CPPPNBs have been utilized for the electrostatic incorporation of fibroblast growth factor 21 (FGF21), forming the complex CPPPNBs@FGF21. This complex has been shown to be effectively delivered to myocardial tissue through ultrasound-induced cavitation effects. In diabetic mouse models, the combination of CPPPNBs@FGF21 with low-frequency ultrasound was observed to significantly downregulate the expression of atrial natriuretic peptide (ANP) and caspase-3, while effectively preventing myocardial hypertrophy, apoptosis, and interstitial fibrosis (86).

However, several limitations remain to be addressed. The precise mechanisms underlying ultrasound-mediated drug delivery have not been fully elucidated. Moreover, potential adverse effects, particularly thermal skin burns, continue to pose significant challenges for clinical implementation.

#### 3.2.2 The microneedle

Microneedles have been developed as a novel drug delivery system that enables direct administration of therapeutic agents into the circulatory system and capillary network for efficient absorption (87). This technology has been demonstrated to enhance drug delivery efficiency while minimizing injection-associated pain (88). In microneedle systems, drugs are typically coated on the surface, facilitating rapid absorption upon skin penetration. Furthermore, dissolvable microneedles have been fabricated from biocompatible pharmaceutical materials, allowing them to be completely absorbed by the body after drug administration. Hollow microneedles have been designed to encapsulate medications within their chambers, enabling controlled release into the dermal tissue. Recent advancements have led to the development of microneedle patch systems, which are combined with various types of transdermal patches for enhanced functionality (87, 89–92). Clinical efficacy has been demonstrated in the treatment of androgenic alopecia, where minoxidil is delivered through microneedles combined with ozone water, showing superior results compared to conventional delivery methods (93). An innovative approach involves the construction of a dissolvable microneedle patch containing FGF21, which has been shown to significantly improve therapeutic efficiency. The optimized design allows for easy skin insertion and penetration, with approximately 38.50 ± 13.38% of FGF21 being released within the first 10 min after application (94). This system has been proven to facilitate FGF21 delivery through the skin barrier while maintaining sustained drug release. Additionally, microneedle technology has been successfully utilized for the efficient loading and transdermal delivery of various pharmaceutical compounds. Through this approach, drugs are effectively delivered into the dermal layer, promoting their penetration through the skin barrier and subsequent systemic absorption.

In addition to ultrasound and microneedle technologies, other physical enhancement methods have been widely employed in TDDS. Thermal ablation and electroporation are among the most extensively studied physical approaches, each offering unique mechanisms for overcoming the skin barrier. Simultaneously, chemical enhancement methods, particularly microvesicles and nanoemulsions, have demonstrated significant potential in TDDS applications. These chemical strategies have been developed to modify the physicochemical properties of drugs and optimize their permeation through the stratum corneum.

### 3.3 The chemical methods

Chemical enhancement methods in TDDS are primarily characterized by the utilization of synthetic mixtures containing transdermal penetration enhancers combined with active pharmaceutical ingredients. These formulations are specifically designed to optimize the percutaneous absorption rate and therapeutic efficacy of administered drugs (95). The chemical enhancement approach is particularly effective for pharmaceutical compounds that exhibit specific physicochemical properties: low molecular weight (<500 Da), appropriate lipophilicity (logP value between 1 and 3), limited skin irritation potential, and relatively short biological half-life. The research focus in transdermal technology has progressively evolved, transitioning from the development of traditional chemical penetration enhancers to the integration of advanced physical enhancement strategies. Contemporary TDDS approaches aim to enhance drug permeation through innovative mechanisms, including the optimization of drug diffusion coefficients and solubility parameters in the stratum corneum. This has been achieved through the development of advanced drug delivery vehicles such as microemulsions, nanoemulsions, and novel vesicular systems, including liposomes, niosomes, and transfersomes (96). These technological advancements have significantly expanded the range of therapeutic agents that can be effectively administered through the transdermal route.

#### 3.3.1 Microvesicles

Microvesicles, defined as colloidal particles containing aqueous cores enclosed by bilayered structures of parent molecules, have been developed as versatile drug carriers for transdermal applications. These vesicular systems are capable of transporting both hydrophilic and lipophilic drugs across the skin barrier into capillary networks, thereby facilitating efficient transdermal absorption (97). In TDDS, the multilamellar architecture of vesicles has been utilized to precisely modulate drug absorption kinetics, enabling sustained drug release following topical application.

Vesicular systems are classified into distinct categories based on their chemical composition, with the main types being liposomes, transfersomes, and ethosomes (98). Liposomes, characterized by their spherical morphology and flexible bilayer membrane structure, have been extensively studied for dermal applications. Although liposomes are unable to penetrate beyond the stratum granulosum layer and remain localized on the skin surface, this limitation has been strategically utilized to prolong drug retention in the epidermis while minimizing systemic absorption. This unique distribution pattern has been exploited to achieve prolonged and sustained drug release profiles (99).

A specific lipid nanoparticle formulation (LNP) has been engineered for the encapsulation and delivery of fibroblast growth factor 21 (FGF21). These FGF21-containing LNPs have been demonstrated to promote sustained release of the therapeutic protein in anti-inflammatory applications (100). While the utilization of liposomal systems for FGF21 delivery remains relatively unexplored, this approach has been recognized as a promising strategy for optimizing the therapeutic efficacy of FGF21 in various clinical applications.

#### 3.3.2 Nanoemulsions

Nanoemulsions have been established as a novel category within nano-drug delivery systems, characterized by their stable, transparent, and low-viscosity properties, which are achieved through precise formulation of water phases, oil phases, surfactants, and co-surfactants in specific proportions (101). The barrier properties of the stratum corneum have been recognized as a significant limitation for transdermal drug delivery, often resulting in reduced absorption efficiency and diminished therapeutic outcomes. Nanoemulsion technology has been developed to overcome this limitation through its inherent low surface tension, which enhances skin wettability and modifies the stratum corneum structure. This modification has been shown to increase lipid bilayer fluidity, disrupt aqueous channels, and reduce skin barrier function. Furthermore, nanoemulsions have been demonstrated to interact effectively with skin cells, facilitating the transdermal absorption of low-molecular-weight drugs while minimizing cutaneous irritation and enabling sustained drug release (102).

The sulconazole nanoemulsion has been successfully prepared using the spontaneous titration method. *In vitro* permeation studies have revealed that the nanoemulsion formulation exhibits 3-fold higher flux and 1.7-fold greater cumulative permeability compared to conventional miconazole cream (103). These results have conclusively demonstrated the enhanced skin permeability characteristics of nanoemulsion-based delivery systems.

Innovative nanoparticle technology has been developed through the utilization of amino-functionalized and embedded dual-mesoporous silica nanoparticles (N-EDMSNs) as carriers for FGF21 plasmid delivery in murine models. When compared to standard FGF21 injection, the N-EDMSNs-pFGF21 treatment has been shown to achieve superior hepatic FGF21 expression while significantly reducing food intake, body weight, and blood glucose levels (104).

### 3.4 Biological methods

Cell-penetrating peptides (CPPs), alternatively referred to as protein transduction domains or membrane-penetrating peptides, were first implemented as agents for transdermal delivery (105). These peptides are principally composed of cationic or amphipathic membrane-interactive sequences that demonstrate the capacity to traverse cellular membranes (106). The category of CPPs has been extended to include protein transduction domains, arginine-rich peptides, bioportides, and numerous other peptide sequences as identified through experimental and computational predictions (107).

The utility of CPPs has been demonstrated in the delivery of diverse therapeutic agents, including but not limited to fluorophores, nucleic acids, peptides, and peptide nucleic acids (108–110). It has been well established that extracellular proteins and peptides exhibit inherent impermeability to cellular membranes, necessitating the implementation of delivery vectors for intracellular transport, as exemplified by the requirement for delivery systems for therapeutic proteins (111).

The association of proteins or peptides with CPPs is achieved through multiple strategies: chemical cross-linking, genetic fusion through cloning and expression of CPP-conjugated proteins, or through non-covalent interactions (112, 113). The enhancement of peptide internalization by CPPs has been substantiated in various studies, including the improved cellular uptake of glucagon-like peptide-2 following conjugation with CPP R8 (114). Similarly, the translocation efficiency of reporter proteins, such as EGFP, has been significantly enhanced using CPPs like Pas2r12 (115). This methodology has been successfully extended to other reporter and model proteins of varying molecular weights and physicochemical properties, including *β*-galactosidase.

TD1 (ACSSSPSKHCG), a transdermal peptide identified through phage display screening technology, has been demonstrated to facilitate the targeted delivery of therapeutic proteins, including insulin, growth hormone, and human epidermal growth factor (hEGF), to the deep dermal layers via the follicular pathway (116–118). When TD1 is genetically fused with hEGF, not only is the biological activity of hEGF preserved, but its transdermal delivery efficiency is also significantly enhanced (119). These experimental observations have confirmed the efficacy of TD1 as a potent transdermal delivery enhancer for therapeutic proteins.

A short peptide designated as SPACE has been employed in phage display screening technology to identify its transdermal delivery capabilities. Through systematic *in vitro* transdermal studies, SPACE has been demonstrated to form stable conjugates with both small molecules and proteins, facilitating their penetration through the dermal layers within 30 min and enabling their delivery to deep dermal capillaries within 2 h, thereby promoting systemic absorption of therapeutic agents (120).

A novel delivery complex, SE-siRNA, has been developed through the conjugation of SPACE with EGF, which has been established as an effective carrier for siRNA delivery across the stratum corneum into viable cells, subsequently inducing programmed cell death in malignant cells (121). The delivery efficiency of the SE-siRNA complex has been shown to significantly exceed that of control siRNA formulations, with the enhanced transdermal penetration capacity being attributed to the SPACE-mediated transport mechanism (122).

The development of transdermal peptides has been recognized as an effective solution to the challenges of poor drug absorption caused by restrictive skin barrier properties in TDDS. These peptides have been demonstrated to significantly enhance the bioavailability of macromolecular drugs. When compared to physical and chemical enhancement methods, biological transdermal strategies have been characterized by milder application conditions and superior preservation of protein bioactivity, while eliminating the potential risks of skin tissue damage associated with alternative methods. The biological transdermal delivery system has been identified as a promising research direction for advancing the therapeutic development and clinical application of FGF21.

For a comprehensive overview of the benefits and potential drawbacks of these approaches, please refer to Table 1, which summarizes the key aspects of each method.

**Table 1:** Classification of implementation methodologies in transdermal drug delivery.

Classification	Implementation	Advantages	Shortcomings
Physical approach	Ultrasound-induced penetration	1. High-frequency ultrasound (20 kHz–16 MHz) creates aqueous channels, boosting drug penetration. 2. Independent of the drug’s physicochemical characteristics, including solubility, dissociation constant, and hydrophilicity	1. Results in cutaneous thermal damage 2. Demonstrates restricted clinical translatability
	Microneedle	1. Painless, efficient delivery directly to dermal capillaries 2. Designable as diverse forms: soluble and hollow types	1. Limited drug loading capability 2. Complex manufacturing, higher costs
Chemical methods	Microvesicles	1. Carries both hydrophilic and hydrophobic drugs simultaneously 2. Modulates drug release kinetics to enhance epidermal retention duration	1. Limited penetration 2. Poor stability
	Nanoemulsions	1. Enhances cutaneous hydration capacity 2. Exhibits transparency coupled with low viscosity characteristics	1. Complex production process 2. May cause skin irritation
Biological methods	Cell-penetrating peptides	1. Facilitates highly efficient delivery of macromolecular therapeutics 2. Preserves drug bioactivity 3. Gentle and non-invasive 4. Facilitates optimal penetration and localization within the deep dermis	1. Complex synthesis and conjugation technology 2. Causes drug off-target effects
	Specific transdermal peptide	1. Active retention 2. Tissue protection	1. Time- and resource-intensive 2. Macromolecular conjugation may affect structure–activity

### 3.5 New research progress on FGF21 fusion protein

FGF21, as a newly identified metabolic regulator, is primarily expressed in hepatic and adipose tissues where it has been shown to play a crucial role in metabolic regulation. However, its therapeutic potential for metabolic disorders has been limited due to insufficient expression levels in pancreatic *β*-cells. To address this limitation, a recombinant FGF21 construct containing a protein tag has been developed through protein fusion labeling technology. The incorporation of a protein tag has been demonstrated to significantly enhance both the production yield and solubility of FGF21. Furthermore, the utilization of this recombinant FGF21 protein has been shown to simplify purification processes and improve the overall protein purity, thereby facilitating its potential therapeutic applications.

Histidine tags, typically composed of six histidine residues in tandem, are extensively utilized for the purification of recombinant proteins. These tags can be incorporated at either the N-terminal or C-terminal position of target proteins without significant disruption to their structural integrity or biological function. In a recent study, a histidine-tagged smGFP-FGF21 fusion protein was successfully engineered and expressed in Nicotiana benthamiana. The recombinant protein was found to be predominantly expressed in its soluble form, facilitating efficient purification through Ni-NTA affinity chromatography. Notably, protein yields of up to 2 mg per gram of fresh leaves have been achieved using this expression system (123).

The small ubiquitin-like modifier (SUMO) tag, an approximately 11 kDa protein encoded by the yeast Smt3 gene (124), is frequently employed for monitoring the intracellular localization of recombinant proteins. When attached at the N-terminus of target proteins through genetic fusion, the SUMO tag has been demonstrated to facilitate proper protein folding. Furthermore, the hydrophobic core of the SUMO tag has been shown to confer proteolytic stability to the fusion protein. In recent studies, FGF21 has been successfully fused with the SUMO tag using overlapping PCR technology, and the resultant fusion protein has been expressed in *Escherichia coli*. Through IPTG induction, SUMO-FGF21 expression levels have been observed to reach approximately 30% of total cellular protein, with the soluble fraction accounting for over 95% of the expressed protein (125).

Maltose-binding protein (MBP), a macromolecular protein tag, has been characterized by its ability to adopt both open and closed conformational states, which have been demonstrated to exhibit distinct binding properties. When in the open conformation, the MBP tag has been shown to specifically interact with gel resin, while the closed conformation has been found to prevent such binding interactions (126). The solubility of FGF21 has been significantly enhanced through fusion with the MBP tag, which has been observed to reduce the accumulation of FGF21 in inclusion bodies during expression in *Escherichia coli*. FGF21 sequestered in inclusion bodies has generally been considered non-functional due to its lack of biological activity. Notably, the MBP tag has been demonstrated to maintain FGF21 solubility at 37°C under culture conditions, with approximately 8.1 mg of pure FGF21 having been obtained from a 500 mL culture after tag removal (127). The MBP fusion strategy has been conclusively shown to improve FGF21 solubility and substantially facilitate the purification process of recombinant FGF21, making it a valuable tool in protein production and purification workflows.

The fusion of FGF21 with protein tags has been demonstrated to significantly enhance the solubility of the expressed protein, thereby establishing a foundation for optimized purification processes, increased production yields, and reliable protein production protocols. The functional benefits of protein fusion technology have been shown to extend beyond simplifications in purification and solubility enhancement.

Through fusion strategies, target proteins have been observed to exhibit enhanced functional characteristics. Furthermore, fusion proteins have been demonstrated to acquire several advantageous properties, including controlled release through biological barriers, targeted transport mechanisms, and structural stabilization. These properties have been found to significantly improve the pharmacological and biological performance of therapeutic proteins.

## 4 Conclusion

FGF21, a crucial metabolic regulator involved in energy homeostasis modulation, has been extensively studied for its therapeutic potential. Its analogues, including pegozafermin (128) and efruxifermin (129), have been evaluated in clinical trials for the treatment of various metabolic disorders, including diabetes and psoriasis. Current research has expanded the therapeutic applications of FGF21 beyond metabolic diseases to include the treatment of diabetes-induced skin lesions and the promotion of wound healing, revealing novel pharmacological properties. These discoveries have opened new research avenues for FGF21 in addressing diabetic foot complications and skin wound healing, while also suggesting unexplored therapeutic potentials. However, the clinical application of FGF21 has been limited by its short biological half-life and rapid metabolism, often preventing the achievement of threshold therapeutic concentrations.

To address these limitations, innovative approaches involving the conjugation of FGF21 with CCPs and its delivery through TDDSs have been developed. These strategies have been demonstrated to achieve controlled release of FGF21, maintaining therapeutic concentrations within the optimal window while facilitating the penetration of FGF21 and other macromolecules through the skin’s epidermal barrier.

This review explores the potential applications of FGF21 as a novel metabolic regulator, including its research progress in wound repair and the delivery strategies developed to enhance its transdermal efficiency. Given its pivotal role as a metabolic regulator, future research directions should focus on three key objectives: (1) enhancement of FGF21 bioavailability, (2) extension of its therapeutic window, and (3) expansion of its administration routes and clinical applications, thereby maximizing the therapeutic potential of this critical metabolic factor. It should be noted that FGF21 has not yet been approved for wound repair, but its mechanisms and therapeutic potential warrant further in-depth investigation.
